# *In Vitro* Action of Flavonoids in the Canine Malignant Histiocytic Cell Line DH82

**DOI:** 10.3390/molecules181215448

**Published:** 2013-12-12

**Authors:** Gabriel Silva, Ana Lúcia Fachin, Renê O. Beleboni, Suzelei C. França, Mozart Marins

**Affiliations:** Biotechnology Unit, University of Ribeirão Preto, Ribeirão Preto 14096-900, SP, Brazil; E-Mails: biel-189@hotmail.com (G.S.); afachin@unaerp.br (A.L.F.); rbeleboni@unaerp.br (R.O.B.); sfranca@unaerp.br (S.C.F.)

**Keywords:** canine cancer, cytotoxicity, DNA damage, flavonoids, topoisomerase II

## Abstract

Cancer is commonly diagnosed in dogs over the age of 10 and is a leading cause of death due to the lack of effective drugs. Flavonoids possess antioxidant, anti-inflammatory and anticarcinogenic properties and have been studied as chemopreventive agents in human cancer therapy. However, the literature on dogs is sparse. In this study, we analyzed the effect of nine flavonoids on cell viability, DNA damage and topoisomerase IIa/IIb gene expression in a canine tumor cell line (DH82). Apigenin, luteolin, *trans*-chalcone and 4-methoxychalcone showed the highest degree of cytotoxicity in the absence of considerable DNA damage, whereas genistein exhibited low cytotoxicity but induced a high level of DNA damage. These five flavonoids inhibited topoisomerase IIa and IIb gene expression to variable extents and with variable specificity. Genistein exerted a lower inhibitory effect on the two topoisomerases than luteolin and apigenin. *trans*-Chalcone and 4-methoxychalcone exerted greater inhibition of topoisomerase IIa expression than topoisomerase IIb. The differences in the effects between genistein and luteolin and apigenin might be explained by the position of ring B, whereas the more specific effect of chalcones on topoisomerase IIa might be due to their open chain structure.

## 1. Introduction

Dogs have accompanied their owners in the increase in life expectancy as a result of advances in veterinary practice, pet food and veterinary medicine. Unfortunately, as seen in humans, the incidence of cancer has increased among dogs, especially among older animals. It is estimated that one in every 3–4 dogs will develop some form of cancer during their lives, twice as much as in humans [1,2,3]. Despite advances in oncology that have permitted successful treatment, cancer continues to be a leading cause of death in humans and animals. In this respect, alternative approaches are needed to change this scenario and the use of dietary flavonoids as chemopreventive and chemotherapeutic agents is gaining attention for human cancers, but is still overlooked for dogs [1,4].

Flavonoids are a diverse class of polyphenolic compounds produced by plants, which can be divided into three main groups: flavones, flavanones (2,3-dihydroflavones), and isoflavones. These groups differ in structure and ring substitutions [5]. Flavonoids have been indicated as important components of the human diet that contribute to the prevention of heart disease, neurodegenerative diseases, diabetes, and cancer [6,7,8]. The main property of flavonoids is their antioxidant effect, which is due to their ability to chelate metal ions and to sequester and inactivate free radicals [9,10]. However, studies using cell and animal models indicate that flavonoids can also act as inhibitors or inducers of a variety of cellular processes, including the inhibition of growth factor signaling pathways [11,12] and enzyme activity [13,14] and induction of tumor suppressor genes [15,16,17], apoptosis [18], and DNA damage [19,20,21,22], in addition to promoting alterations in gene expression by epigenetic mechanisms [23,24,25]. Moreover, in the presence of metals such as copper and iron, flavonoids increase the formation of free radicals and act as pro-oxidants, causing DNA oxidation, with consequent genotoxic and mutagenic effects including DNA double-strand breaks [26]. This property can be exploited against tumor cells since these cells contain a higher concentration of intracellular copper, especially bound to DNA, thus increasing the genotoxic effect of flavonoids [27].

Flavonoids have also been reported to inhibit topoisomerase II [28], a ubiquitous enzyme with two isoforms in mammals, topoisomerase IIa and IIb. These enzymes promote transient DNA breaks during the processes of chromosome segregation, transcription, and DNA replication. 

The availability of canine tumor cells is an alternative to test new antitumor drugs; however, apart from genistein which has been shown to induce apoptosis in canine lymphoma cell lines, there are few *in vitro* studies supporting the use of flavonoids as chemopreventive or chemotherapeutic agents in dogs [1,29]. The DH82 cell line was established from the neoplastic progenitor cells of a dog with canine malignant histiocytosis [30]. This disease has a poor prognosis in dogs and is characterized by a rapid clinical progression which often results in death. Chemotherapy is a therapeutic alternative, but success rates are still low [31]. Therefore, we investigated the effects of nine flavonoids on the cell viability, DNA damage and topoisomerase IIa and IIb gene expression in DH82 cells in order to increase the knowledge of the effects of flavonoids in this canine tumor cell line. 

**Figure 1:**
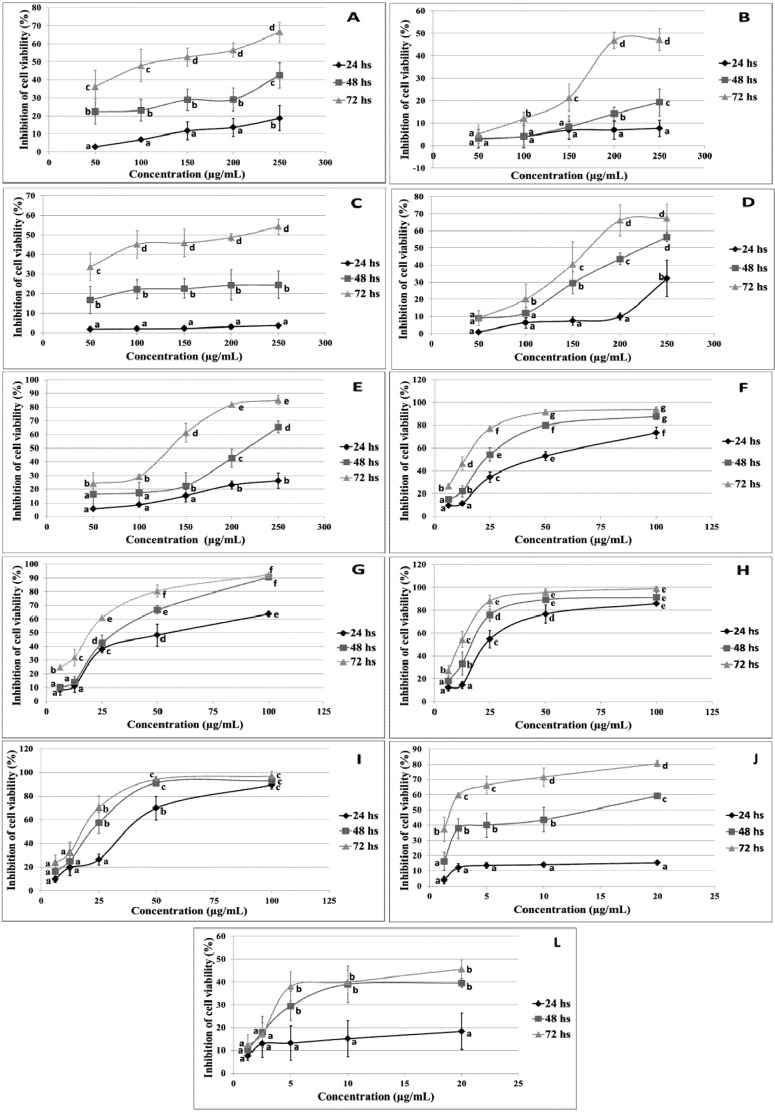
Inhibition of the viability of DH82 cells induced by flavonoids and topoisomerase after 24, 48 and 72 h of treatment. (**A**) Quercetin; (**B**) myricetin; (**C**) fisetin; (**D**) epigallocatechin gallate (EGCG); (**E**) genistein; (**F**) luteolin; (**G**) apigenin; (**H**) trans-chalcone; (**I**) 4-methoxychalcone; (**J**) etoposide; (**L**) merbarone. Symbols represent the mean of at least three independent experiments and the error bar indicates the standard deviation. Different letters indicate statistically significant results (*p* < 0.05).

## 2. Results and Discussion

### 2.1. Cytotoxic Effect of Flavonoids

Studies have shown that flavonoids exert cytotoxic activity against cancer cells, a property that can be explored for the development of new cancer therapies [32]. In an attempt to extend these therapies to dogs, we evaluated the cytotoxic effects of flavonoids in the canine malignant histiocytosis cell line DH82. The MTT assay was used to evaluate the viability of cells treated with different concentrations of the flavonoids and topoisomerase inhibitors for 24, 48 and 72 h. As can be seen in Figure 1, all substances reduced cell viability in a dose-dependent fashion, but with marked differences. Luteolin, apigenin, *trans*-chalcone and 4-methoxychalcone significantly reduced cell viability at lower concentrations within 24 h of treatment, whereas the other flavonoids were less effective after the same period of treatment.

The IC_50_ values obtained for the different flavonoids could be divided into two groups (Table 1). Quercetin, genistein, epigallocatechin gallate (EGCG), myricetin, and fisetin exhibited IC_50_ values higher than 200 µM after 72 h of treatment, or 600 µM after 24 h of treatment. Lower IC_50_ values were observed for apigenin, luteolin, *trans*-chalcone and 4-methoxychalcone, ranging from 52.8 µM at 72 h of treatment (luteolin) to 165.4 µM at 24 h (apigenin). *trans*-Chalcone was the most effective flavonoid after 24 h of treatment (IC_50_: 129.2 µM), whereas luteolin was the most effective flavonoid after 48 and 72 h of treatment (IC_50_: 87.7 and 52.8 µM, respectively). The IC_50 _values obtained for luteolin, apigenin, *trans*-chalcone and 4-methoxychalcone were comparable to that of the topoisomerase inhibitor merbarone. Although quercetin, genistein, fisetin, EGCG and myricetin showed low cytotoxicity in the DH82 cell line, several studies have demonstrated their antiproliferative activity against different cell lines [33,34,35]. This reflects the cell-specific effect of flavonoids which is influenced by the capacity of each cell type to absorb, inactivate or metabolize these substances to more toxic metabolites [36,37,38].

**Table 1:** IC_50_ values of flavonoids and topoisomerase inhibitors in DH82 cells.

Flavonoids and Drugs	IC_50_ (µM)		
	24 h	48 h	72 h
Luteolin	156.2	87.7	52.8
Apigenin	165.4	124.3	79.5
4-Methoxychalcone	154.0	92.7	76.4
*trans*-Chalcone	129.2	89.3	59.5
Quercetin	>992.5	>661.7	>302.2
Genistein	>1110	>740.1	>370
EGCG	>654.5	>436.6	>218.2
Myricetin	>942.7	>628.5	>628.5
Fisetin	>1048	>1048	>349.5
Etoposide	95.5	19.1	3.6
Merbarone	205.5	77.8	65.3

### 2.2. Analysis of the Relationship between Structure and Cytotoxic Effect

**Figure 2:**
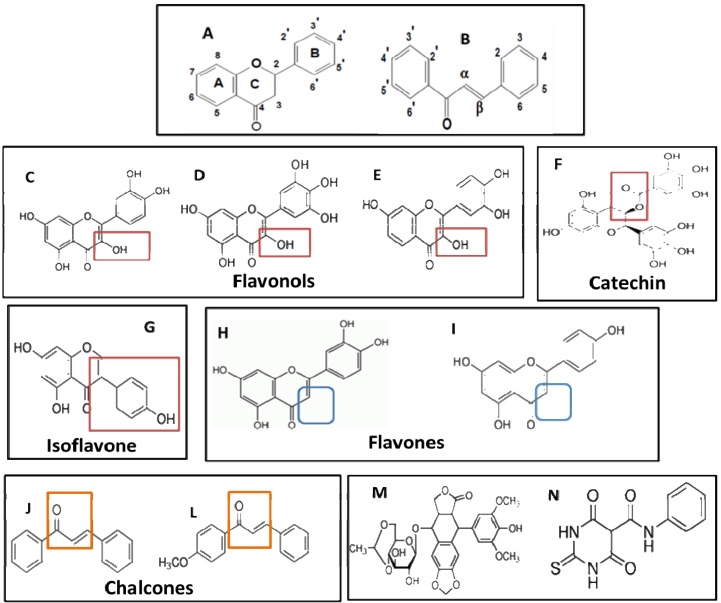
Chemical structure of flavonoids and topoisomerase inhibitors. (**A**) General structure of flavonoids; (**B**) general structure of chalcones; (**C**) quercetin; (**D**) myricetin; (**E**) fisetin; (**F**) epigallocatechin gallate (EGCG); (**G**) genistein; (**H**) luteolin; (**I**) apigenin; (**J**) *trans-*chalcone; (**L**) 4-methoxychalcone; (**M**) etoposide; (**N**) merbarone.

The biological activity of flavonoids has been suggested to be correlated with the position, number and substitution of the hydroxyl group in the A and B rings [39]. In this study, the differences in the cytotoxic effect and IC_50_ values of the flavonoids permitted a preliminary attempt to establish a structure-cytotoxic effect relationship. In all treatments, the IC_50_ values of the two flavonols luteolin and apigenin were comparable to that of the topoisomerase inhibitor merbarone, whereas the flavones quercetin, myricetin and fisetin exhibited values higher than 200 µM. The main difference between flavones and flavonols is the absence of a 3-hydroxyl group at position 3 of the C ring. In genistein, the B ring is attached to this position, whereas in EGCG an extra ring (galloyl-D-ring) is attached to it. Luteolin differs from apigenin by the presence of a 3-hydroxyl at position 3’ of the B ring, but has only a slightly superior cytotoxic effect (Table 2 and Figure 2). Therefore, a group attached to position 3 of the C ring seems to be the most important structural feature for the cytotoxic effect of these flavonoids in the DH82 cell line.

**Table 2:** Structural differences (number and position of substituents) between the nine flavonoids tested in this study.

Flavonoids	Class	Substituents			Total OH	Total COH_3_
		Chain A	Chain B	Chain C		
**Cytotoxic compounds**						
*trans*-Chalcone	Chalcone				0	0
4-Methoxychalcone	Chalcone	COH_3_-4'			0	1
Luteolin	Flavone	OH-5,7	OH-3',4'	O-4; anel-2	4	0
Apigenin	Flavone	OH-5,7	OH-4'	O-4; anel-2	3	0
**Low cytotoxicity compounds**						
Quercetin	Flavonol	OH-,5,7	OH-3',4'	O-4; OH-3; anel-2	5	0
Myricetin	Flavonol	OH-,5,7	OH-3',4',5'	O-4; OH-3; anel-2	6	0
Fisetin	Flavonol	OH-7	OH-4',5'	O-4; OH-3; anel-2	4	0
EGCG	Catechin	OH-5,7	OH-3',4',5'	galato-3; anel-2	8	0
Genistein	Isoflavone	OH-5,7	OH-4'	O-4; anel-3	3	0

The two chalcones tested also exhibited lower IC_50_ values, which were comparable to that of the topoisomerase IIa inhibitor merbarone. After 24 h of treatment, the order of cytotoxicity based on the IC_50_ values of the most effective flavonoids was *trans*-chalcone > 4-methoxychalcone > luteolin > apigenin, while at 48 and 72 h the order was luteolin > *trans*-chalcone > 4-methoxychalcone > apigenin (Table 1). The main difference of chalcones compared to flavones and to the other flavonoids tested is the absence of the heterocyclic C ring (Figure 2). Again, the C ring seems to play a key role in the cytotoxic effects observed. *trans*-Chalcone and 4-methoxychalcone differ in the position of the double bond in the open C ring and in the presence of a methoxy group attached to position 4 of the A ring in 4-methoxychalcone (Figure 2). These differences may explain the lower IC_50_ value observed for *trans*-chalcone.

The presence of a catechol group (3',4'-OH) in the B ring is considered a prerequisite for cytotoxic activity [40]. The positive influence of dihydroxylation of carbons 3 and 6 has also been demonstrated [41]. However, the present results indicate that a substituent at position 3 of the C ring reduced the cytotoxicity of the flavonoids tested. A reduction in cytotoxic activity when a hydroxyl group is present at position 3 of the C ring has been demonstrated [36]. This feature is found in quercetin, myricetin and fisetin, which exhibited very low cytotoxicity against the DH82 cell line. Luteolin and apigenin lack a substituent at position 3 of the C ring and were more cytotoxic than the former. Moreover, *trans*-chalcone and 4-methoxychalcone have an open C ring and both lack a hydroxyl group in their structure. We postulate that the absence of the hydroxyl group near the ketone in ring C is an important feature for the cytotoxic activity of flavonoids, whereas the presence of substituents in the A and B rings has a modulatory effect on cytotoxicity.

### 2.3. Genotoxic Effect

We also investigated the induction of DNA damage by flavonoids in the DH82 cells using the comet assay in order to determine whether the cytotoxicity of the flavonoids studied is directly related to genotoxicity. Most flavonoids showed a DNA damage index (DDI) of 1 or lower (Table 3), indicating low genotoxicity in DH82 cells. Only genistein exerted a significant dose- and time-dependent genotoxic effect, with a DDI of up to 2.79 (Table 3). The formation of comets with defined tails (long and intense) is strongly associated with the stabilization of topoisomerase-DNA cleavage complexes [42]. This is the mechanism of action of etoposide (positive control), thus explaining the high genotoxicity of this topoisomerase inhibitor (DDI 3.22 ± 0.065 after 24 h) when compared to merbarone (DDI 1.14 ± 0.088 after 24 h), which does not stabilize topoisomerase II-DNA cleavage complexes. It was therefore suggested that merbarone induces less DNA damage than etoposide [43], a fact confirmed in the present study using DH82 cells (Table 3). Greater stabilization of the DNA-topoisomerase cleavage complex is a major determinant of DNA damage, since it increases the chance that the complex collides with the replication and transcription machinery [44]. Genotoxic effects mediated by this mechanism have been reported for several flavonoids [45,46,47], including those tested here [45,46,47]. However, the only flavonoid that induced significant DNA damage in the present study was genistein (Table 3). In contrast, this flavonoid did not induce significant cytotoxicity at the same concentration or after the same time of treatment. Similarly, etoposide, a topoisomerase inhibitor, induced low cytotoxicity (Figure 1), but high DNA damage (Table 3), when the cells were treated with a concentration of 2.5 µg/mL for 24 h. Similar results have been reported in other studies [42]. Quercetin, EGCG, myricetin and fisetin did not induce significant cytotoxicity or genotoxicity, suggesting low specificity of these flavonoids for DH82 cells. Luteolin and apigenin also did not induce DNA damage, but were moderately cytotoxic, indicating that low genotoxicity is not related to low specificity for DH82 cells. The two chalcones tested were cytotoxic, but did not induce elevated levels of DNA damage. There are few studies investigating the biological effects of these substances and it is unlikely that their cytotoxic effects are due to genotoxicity induced by topoisomerase poisoning. Topoisomerase II poisoning can occur by redox-independent and redox-dependent mechanisms. In the first mechanism, flavonoids with a closed chain require the formation of a pseudo-ring between the ketone group of the C ring and a close hydroxyl (OH at position 5). In redox-dependent poisoning, the presence of a pyrogallol (OH at positions 3', 4' and 5') is necessary, whose oxidation leads to the formation of hydrogen peroxide, inducing the formation of topoisomerase-DNA complexes [44,48]. Chalcones have an open C ring and those tested in this study do not contain any hydroxyl group (Figure 2 and Table 2), supporting the possibility that they do not induce DNA damage as demonstrated by the comet assay, especially not through topoisomerase poisoning.

**Table 3:** Genotoxicity of flavonoids and topoisomerase inhibitors in DH82 cells.

Genotoxicity			
Substances	Concentration (µg/mL)	DNA Damage Index: 0–4	
		24 h	6 h
**Control (0.5% DMSO)**		0.36 ± 0.070 a	0.4 ± 0.061 a
**Etoposide**	2.5	3.22 ± 0.065 h	2.5 ± 0.115 f
**Merbarone**	2.5	1.14 ± 0.088 d	0.79 ± 0.052 c
**Genistein**	200	2.79 ± 0.067 g	1.84 ± 0.17 e
	100	1.80 ± 0.083 e	1.15 ± 0.105 d
	50	1.10 ± 0.065 d	0.83 ± 0.095 c
**Quercetin**	200	0.65 ± 0.094 b	-
**EGCG**	200	1.03 ± 0.031 d	-
**Myricetin**	200	0.88 ± 0.055 c	-
**Fisetin**	200	0.83 ± 0.032 c	-
**Luteolin**	25	0.74 ± 0.066 b	-
	12.5	0.68 ± 0.075 b	-
***trans*-Chalcone**	25	0.64 ± 0.051 b	0.51 ± 0.079 a
	12.5	0.57 ± 0.043 a	0.50 ± 0.039 a
**4-Methoxychalcone**	25	0.73 ± 0.085 b	0.48 ± 0.062 a
	12.5	0.66 ± 0.074 b	0.47 ± 0.056 a
**Apigenin**	25	0.80 ± 0.063	0.66 ± 0.096 b
	12.5	0.74 ± 0.052 b	0.57 ± 0.107 a

DNA damage index: 0 (no DNA damage) to 4 (total DNA damage). The results are expressed as the mean ± standard error of at least three independent experiments. Different letters indicate statistically significant results (*p* < 0.05).

### 2.4. Effect of Flavonoids on the Expression of Topoisomerase II

**Figure 3:**
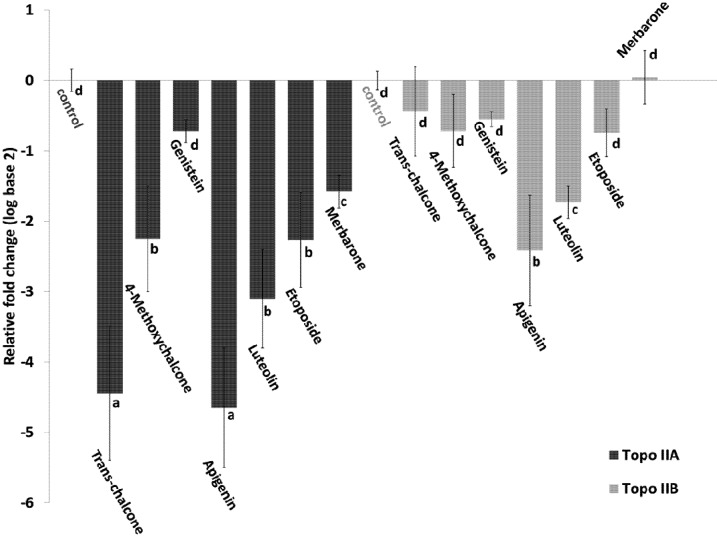
Effect of flavonoids and topoisomerase inhibitors on the expression of topoisomerase IIa and IIb genes. The cells were treated with flavonoids at a concentration of 12.5 µg/mL for 6 h. The concentration of etoposide and merbarone was 2.5 µg/mL. The results are expressed as the mean ± standard error of at least three independent experiments. Different letters indicate statistically significant results (*p* < 0.1).

The effect of *trans*-chalcone, 4-methoxychalcone, luteolin, apigenin, genistein and the two topoisomerase inhibitors on the expression of the topoisomerase IIa and IIb genes in DH82 cells was evaluated by quantitative RT-PCR. Genistein was chosen for analysis because it was the most genotoxic flavonoid, while the other flavonoids were the most cytotoxic. As shown in Figure 3, except for merbarone, all flavonoids and etoposide inhibited the two topoisomerases to variable extents (negative fold change). The repression of topoisomerase IIa was more marked than that of topoisomerase IIb. This finding was more evident for *trans*-chalcone and 4-methoxychalcone, which inhibited topoisomerase IIa ten and three times more than topoisomerase IIb, respectively. The highest levels of repression of the IIa isoform were −4.65 and −4.45 seen in the treatments with apigenin and *trans*-chalcone, respectively. Apigenin exerted the strongest inhibitory effect on the IIb isoform, followed by luteolin.

The cytotoxicity and genotoxicity results might be explained by the repression or expression of specific pathways targeted by the different flavonoids studied. In this respect, many flavonoids induce cell cycle arrest related to topoisomerase II inhibition [35,49,50]. One example is the inhibition of topoisomerase IIa mRNA expression by genistein in HeLa cells, which has been shown to be related to interruption of the cell cycle and induction of apoptosis [51]. In this study, we determined whether flavonoids with significant cytotoxic and genotoxic activity influence DNA topoisomerase IIa and IIb gene expression at the transcriptional level. Luteolin, apigenin, *trans*-chalcone, and 4-methoxy-chalcone inhibited mRNA expression of both topoisomerases II and, more specifically, of the IIa isoform. The repression of topoisomerase IIa induces cell death by mitotic failure as a result of incorrect segregation of the chromosomes to the cell poles during mitosis [52].

The more marked inhibition of the IIa isoform may be advantageous for exploring chalcones in cancer therapies, since the IIb isoform seems to play a key role in the induction of secondary neoplasias [19,53] and in the cardiotoxic effect of drugs such as doxorubicin [54]. Moreover, the inhibition of topoisomerase IIb may compromise DNA repair in neuronal tissue [55,56]. Another fact is that catalytic inhibitors are more effective in cells expressing topoisomerase II at low levels [57]. Therefore, the sensitivity of cancer cell lines to catalytic inhibitors could be increased by combining them with chalcones, thus reducing the use of topoisomerase poisons and, consequently, side effects such as secondary neoplasias.

## 3. Experimental

### 3.1. Materials

The topoisomerase inhibitors merbarone, etoposide, quercetin, *trans*-chalcone, and 4-methoxychalcone were purchased from Sigma-Aldrich (St. Louis, MO, USA). All other flavonoids were purchased from Cayman Chemical (Ann Arbor, MI, USA). (3-(4,5-Dimethylthiazol-2-yl)-2,5-diphenyltetrazolium bromide (MTT), dimethyl sulfoxide (DMSO), Triton X-100, Dulbecco’s modified Eagle’s medium (DMEM), fetal bovine serum, and DNase I were purchased from Sigma-Aldrich. Normal and low-melting point agarose were purchased from USB (Cleveland, OH, USA). NaCl, Tris, NaOH and EDTA were purchased from Synth (Diadema, SP, Brazil). The Illustra RNAspin Mini RNA Isolation kit was purchased from GE Healthcare (Piscataway, NJ, USA). The TaqMan^®^ Gene Expression assay and High-Capacity cDNA Reverse Transcription kit were purchased from Applied Biosystems (Foster City, CA, USA).

### 3.2. Cell Culture

The canine malignant histiocytic cell line DH82 (ATCC number: CRL-10389) [30] was cultured in DMEM supplemented with 10% fetal bovine serum and incubated in a humidified incubator in a 5% CO_2_ atmosphere at 37 °C. In the different assays, DMSO was used as a solvent of the chemical compounds at an atoxic concentration for the cells (0.5% or less). 

### 3.3. Cytotoxicity Assay

The cytotoxicity of the different chemicals was analyzed by the MTT assay [58]. The cells were seeded in 96-well plates at a concentration of 2 × 10^5^ cells/well and cultured for 24 h in a 5% CO_2_ atmosphere at 37 °C. After this period, the chemicals diluted in fresh medium to five different concentrations were added to the wells and the cells were cultured under the same conditions for 24, 48 and 72 h. The medium with the substances was changed at intervals of 24 h. All cell treatments were carried out in triplicate. After each treatment period, the medium was replaced with fresh medium without chemicals, 20 µL MTT solution (5 mg/mL) was added to each well, and the plates were incubated for an additional 4 h under the same conditions. The plates were centrifuged at 3500 × g for 5 min, the supernatant was discarded, and 200 µL DMSO was added to dissolve the formed formazan crystals. Absorbance was read in a Thermoplate reader at a wavelength of 550 nm. The results were plotted as the percentage of inhibition of cell viability (ICV) calculated using the following formula:

ICV (%) = [1 − (average absorbance of experimental group/average absorbance of cells treated with 0.5% DMSO control group)] × 100

### 3.4. DNA Damage Assay

DNA damage was evaluated by the single-cell gel electrophoresis assay (comet assay) [59]. The cells were cultured in 24-well plates at a concentration of 2 × 10^5^ cells/well (5% CO_2_ atmosphere, 37 °C) in the presence of the chemicals at a concentration that permitted ~70% cell viability, for periods of 6 and 24 h. Etoposide and merbarone (2.5 µg/mL) were used as positive controls and 0.5% DMSO as solvent control. After the treatments, the cells were transferred to a microtube and centrifuged at 350 × g for 5 min at 4 °C. The supernatant was discarded and the cell pellet was resuspended in 200 µL low-melting point agarose (0.5% in PBS). The agarose/cell suspension was pipetted onto microscope slides previously coated with low-melting point agarose (1.5% in PBS). The agarose/cell suspension was covered with coverslips and the slides were incubated for 10 min at 4 °C. Three slides were prepared per treatment. After removal of the coverslips, the slides were immersed in lysis buffer (2.5 M NaCl, 100 mM EDTA, 10 mM Tris, 1% Triton X-100, and 10% DMSO) for 20 h at 4 °C in the dark. After rinsing with electrophoresis solution (10 M NaOH, 200 mM EDTA, pH 13) for 20 min at 4 °C in the dark, the slides were submitted to electrophoresis at 0.8 volts/cm for 20 min at 4 °C in the dark. Next, the slides were removed from the electrophoresis chamber and rinsed with neutralization buffer (0.4 M Tris, pH 7.5) for 15 min, air dried at room temperature, and fixed with absolute ethanol for 3 min. The slides were stained with 40 µL of an ethidium bromide solution (0.02 mg/mL) and visualized under a fluorescent microscope. One hundred comets/slide were randomly selected and DNA damage was visually scored based on the length and intensity of the tail into five classes ranging from 0 (no DNA damage) to 4 (maximal DNA damage) [60]. The DNA damage index (DDI) was calculated using the formula:

DDI = (No. of cells in class 0 × 0 + No. of cells in class 1 × 1 + No. of cells in class 2 × 2 + No. of cells in class 3 × 3 + No. of cells in class 4 × 4)/No. of comets analyzed

### 3.5. Quantification of Topoisomerase IIa and Topoisomerase IIb Messenger RNA (mRNA)

The cells were cultured in 24-well plates at a concentration of 2 × 10^6^ cells/well in a 5% CO_2_ atmosphere at 37 °C. After adhesion, the cells were treated for 6 h with the chemicals at a concentration that permitted ~70% cell viability. Next, mRNA was extracted from the cells and purified using the Illustra RNAspin Mini RNA Isolation kit (GE Healthcare). After treatment with DNAse I (Sigma-Aldrich), 1 µg mRNA was used for synthesis of cDNA using the High-Capacity cDNA Reverse Transcription kit (Applied Biosystems). Messenger RNA was quantified in a total volume of 20 μL using the TaqMan^®^ Gene Expression Assay (Applied Biosystems) according to manufacturer instructions. The reaction mixture contained 1 µL 20X TaqMan^®^ Gene Expression Assay (TaqMan^®^ Gene Expression Assay IDs are shown in Table 1), 10 µL 2X TaqMan^®^ Gene Expression Master Mix, 1 µL cDNA, and 8 µL RNase-free water. A negative control was included for each gene and amplification reactions were performed in triplicate in an Mx3005P real-time thermocycler (Stratagene, La Jolla, CA, USA), with one hold at 95 °C for 10 min and 40 cycles of 95 °C for 15 s and 60 °C for 1 min. The fluorescence data were collected at the end of the annealing/elongation step. Data were analyzed and plotted using the MxPro software (Stratagene). The RPL32 gene was used as a reference for normalization of the results and fold-differences in gene expression were calculated relative to control samples (cells treated with 0.5% DMSO) using the ΔΔCT method. 

### 3.6. Statistical Analysis

The data were analyzed by two-way analysis of variance (ANOVA), followed by the Scott-Knott test (*p* < 0.05 or *p* < 0.1). The IC_50_ values were calculated by nonlinear regression analysis from the ICV-concentration data. The results of ICV, DDI and fold-change in gene expression are plotted as the mean ± standard error of at least three biological replicates.

## 4. Conclusions

Although chemotherapy for canine cancer is available, treatment is often palliative and euthanasia is still necessary in most cases. Even at low doses, the commonly used drug doxorubicin can cause cardiomyopathies [61]. Therefore, the search for novel anticancer drugs is also mandatory for dogs. The results of this study raise the possibility of exploring flavonoids for anticancer therapies in dogs, as well as in humans, in view of their lower toxicity when compared to other drugs.
